# Recurrence biomarkers of triple negative breast cancer treated with neoadjuvant chemotherapy and anti-EGFR antibodies

**DOI:** 10.1038/s41523-021-00334-5

**Published:** 2021-09-17

**Authors:** Nina Radosevic-Robin, Pier Selenica, Yingjie Zhu, Helen H. Won, Michael F. Berger, Lorenzo Ferrando, Emiliano Cocco, Maud Privat, Flora Ponelle-Chachuat, Catherine Abrial, Jean-Marc Nabholtz, Frederique Penault-Llorca, Jorge S. Reis-Filho, Maurizio Scaltriti

**Affiliations:** 1 Department of Pathology, Centre Jean Perrin, Clermont-Ferrand, France; 2 University Clermont Auvergne, INSERM U1240 “Molecular Imaging and Theragnostic Strategies (IMoST)”, Clermont-Ferrand, France; 3 Department of Pathology, Memorial Sloan Kettering Cancer Center, New York, NY USA; 4 Human Oncology & Pathogenesis Program (HOPP), Memorial Sloan Kettering Cancer Center, New York, NY USA; 5 Department of Oncogenetics, Centre Jean Perrin, Clermont-Ferrand, France; 6 Department of Clinical Research, Centre Jean Perrin, Clermont-Ferrand, France; 7 Department of Medical Oncology, Centre Jean Perrin, Clermont-Ferrand, France; 8 Present Address: Division of Hematology/Oncology, Department of Medicine, King Khalid University Hospital/College of Medicine, King Saud University, Riyadh, Saudi Arabia; 9 Present Address: AstraZeneca, Waltham, MA USA

**Keywords:** Cancer genomics, Prognostic markers, Transcriptomics, Tumour immunology, Cancer

## Abstract

To find metastatic recurrence biomarkers of triple-negative breast cancer (TNBC) treated by neoadjuvant chemotherapy and anti-EGFR antibodies (NAT), we evaluated tumor genomic, transcriptomic, and immune features, using MSK-IMPACT assay, gene arrays, Nanostring technology, and TIL assessment on H&E. Six patients experienced a rapid fatal recurrence (RR) and other 6 had later non-fatal recurrences (LR). Before NAT, RR had low expression of 6 MHC class I and 13 MHC class II genes but were enriched in upregulated genes involved in the cell cycle-related pathways. Their TIL number before NAT in RR was very low (<5%) and did not increase after treatment. In post-NAT residual tumors, RR cases showed high expression of *SOX2* and *CXCR4*. Our results indicate that high expression of cell cycle genes, combined with cold immunological phenotype, may predict strong TNBC resistance to NAT and rapid progression after it. This biomarker combination is worth validation in larger studies.

## Introduction

Triple-negative breast cancer (TNBC) is characterized by the lack of expression of estrogen receptor (ER) and progesterone receptor (PR) and the absence of amplification of *ERBB2*. It encompasses a large spectrum of breast malignancies and is highly heterogeneous in terms of histology, molecular features, and clinical behavior^1^. Although some of these tumors are characterized by low histological grade and good long-term prognosis after local treatment, the majority present with a high grade and aggressive nature reflected by an early peak of recurrence, fatal distant metastases, and the 5-year overall survival (OS) rate lower than the other subtypes of breast cancer^2,3^. Therefore, finding reliable predictors of life-threatening recurrences is an unmet need for TNBC.

Most TNBC recurrences develop within 5 years after the diagnosis^4^, and it is accepted that the risk of recurrence is higher in TNBC patients with a big residual tumor (RT) after neoadjuvant treatment (NAT)^5^. Balko and collaborators have elegantly shown that post-NAT TNBC RTs carry numerous genomic alterations and that 90% of these tumors have at least one actionable molecular lesion^6^. This study posited that molecular alterations found in the post-NAT residual disease may be the same present in micrometastases that will cause disease recurrence.

Beyond genomic anomalies, recent studies have highlighted the importance of TNBC immunological subtype both for response to NAT and for further clinical evolution. TNBCs rich in tumor-infiltrating lymphocytes (TILs), either before or after NAT, have better outcomes than those poor in TILs^7,8^. Besides TIL quantity, gene expression profiling has revealed the existence of several prognostically relevant TNBC subtypes, with different immune microenvironment composition and immune response within the breast tumor tissue^9,10^.

However, none of those subtypes or other gene signatures that emerged as recurrence predictors in TNBC is sufficiently validated to enter the clinics^11–17^. In addition, no biological marker has been proposed to identify TNBCs that will progress shortly after NAT. Tumor relapses occurring within the first year after NAT are considered manifestations of primary chemoresistance and their long-term favorable outcome is exceedingly rare. In those tumors, chemotherapy can even stimulate metastatic progression by selecting the cells with high stemness and proliferation or by modifying the tumor microenvironment to favorize the survival and multiplication of metastatic tumor cells^18–21^. Therefore, the patients carrying such tumors would benefit from early identification, ideally before NAT, and be offered more advanced therapy approaches than the standard anthracycline-taxane regimens.

On the other hand, real-life observations in the clinic confirm that certain TNBC patients will never experience recurrence, even left with a big RT and no adjuvant chemotherapy after NAT. If readily identified at the post-NAT surgery, this patient population could be included in clinical trials testing de-escalation of adjuvant treatments. However, studies showing how to recognize either the TNBCs which will rapidly progress after NAT or the ones which will remain under long-term control despite big post-NAT residual tumors, are lacking.

Here we report a comprehensive genomic and transcriptomic analysis, integrated with lymphocyte infiltration, of pre-treatment and post-NAT samples from TNBC patients undergoing chemotherapy and EGFR blockade^22,23^.

## Results

### Patient population

We analyzed a total of 62 patients, 12 of them experiencing metastatic recurrences. Six patients recurred within a year after surgery (group A) and rapidly deceased from metastatic disease. Six patients had later recurrences, mostly without a fatal outcome (in 1–5 years after surgery, group B) (Table 1 and Supplementary Fig. 1). Since we were interested in comparing molecular characteristics of the recurring tumors versus those of non-recurring tumors of various sizes, we further classified the non-recurring tumors into 4 groups: group C (8 patients, breast RT size, defined by its greatest dimension, >30 mm); group D (4 patients, RT size ≤30 mm but >20 mm); group E (18 patients, RT size ≤20 mm but not pCR); group F (20 patients, pCR). The only two patients with pCR and a recurrence were integrated into group B (patients 38 and 72, Table 1 and Supplementary Fig. 1). This arbitrary classification was used as we could not determine residual cancer burden in all cases because of a lack of complete breast and lymph node material in some cases treated outside of Centre Jean Perrin (external participants in the trials).

**Table 1:** Patient clinical characteristics.

Pt ID	Group	Trial	Age	TN	Stage	Histological type	Response to Th	Breast RT size (mm)	Number of involved LNs post-NAT	Recurrence site	TTR, from surgery (months)	Survival from surgery until death or the end of follow-up (months)
65	A	PTMB	55	T2N0	IIA	NST	Non-pCR	30	0	Lungs, bone	6	8
49	A	PTMB	55	T3N1	IIIA	NST	Non-pCR	70	9	Liver	3	8
9	A	PTMB	44	T2N0	IIA	NST	Non-pCR	50	1	Brain	5	6
52	A	PTMB	67	T2N0	IIA	NST	Non-pCR	80	6	Breast, lungs	5	7
46	A	CTX	46	T2N0	IIA	NST	Non-pCR	45	0	Lungs	12	17
71	A	PTMB	42	T2N0	IIA	NST	Non-pCR	18	2	Lungs, liver	9	15
38	B	PTMB	50	T3N0	IIB	NST	pCR	0	0	Solitary brain metastasis	40	60
10	B	PTMB	73	T3N2	IIIA	Apocrine	Non-pCR	60	9	Lungs	34	60
33	B	CTX	64	T3N0	IIB	NST	Non-pCR	20	0	Lungs	46	60
44	B	CTX	50	T2N1	IIB	NST	Non-pCR	55		Lungs	17	60
72	B	PTMB	65	T2N0	IIA	NST	pCR	0	0	Brain	23	24
73	B	PTMB	57	T2N0	IIA	NST	Non-pCR	5	1	Lungs, liver	17	26
32	C	PTMB	35	T3N1	IIIA	NST	Non-pCR	45	2	None	NA	60
60	C	CTX	67	T3N1	IIIA	NST	Non-pCR	60	0	None	NA	60
54	C	PTMB	29	T2N0	IIA	NST	Non-pCR	55	1	None	NA	60
23	C	CTX	54	T3N1	IIIA	NST	Non-pCR	50	0	None	NA	60
50	C	PTMB	42	T2N0	IIA	NST	Non-pCR	45	0	None	NA	60
21	C	CTX	61	T2N1	IIB	NST	Non-pCR	35	0	None	NA	60
11	C	PTMB	42	T2N1	IIB	NST	Non-pCR	35	2	None	NA	60
48	C	PTMB	53	T3N2	IIIA	NST	Non-pCR	35	0	None	NA	60
68	D	PTMB	62	T2N1	IIB	NST	Non-pCR	30	1	None	NA	60
34	D	PTMB	44	T2N0	IIA	NST	Non-pCR	25	0	None	NA	60
45	D	CTX	42	T2N0	IIA	NST	Non-pCR	25	0	None	NA	60
56	D	CTX	54	T2N0	IIA	NST	Non-pCR	25	0	None	NA	60
13	E	PTMB	50	T2N0	IIA	NST	Non-pCR	20	0	None	NA	60
69	E	PTMB	40	T3N0	IIB	NST	Non-pCR	20	0	None	NA	60
67	E	PTMB	54	T2N0	IIA	NST	Non-pCR	20	1	Breast	NA	60
64	E	CTX	37	T2N1	IIB	NST	Non-pCR	20	1	None	NA	60
40	E	CTX	59	T2N0	IIA	NST	Non-pCR	20	0	None	NA	60
57	E	CTX	53	T3N0	IIB	NST	Non-pCR	16	0	None	NA	60
70	E	CTX	28	T2N1	IIB	NST	Non-pCR	15	4	None	NA	60
30	E	CTX	47	T3N0	IIB	NST	Non-pCR	12	4	None	NA	60
28	E	CTX	34	T2N1	IIB	NST	Non-pCR	10	0	None	NA	60
36	E	PTMB	39	T3N0	IIB	NST	Non-pCR	9	0	None	NA	60
35	E	CTX	38	T2N2	IIIA	NST	Non-pCR	7	3	None	NA	60
19	E	CTX	52	T3N0	IIB	NST	Non-pCR	2	3	None	NA	60
74	E	PTMB	35	T2N0	IIA	NST	Non-pCR	6	0	None	NA	60
75	E	CTX	60	T2N0	IIA	NST	Non-pCR	3	0	None	NA	60
76	E	PTMB	48	T2N1	IIB	NST	Non-pCR	2	2	None	NA	60
84	E	PTMB	43	T3N1	IIIA	NST	Non-pCR	7	2	None	NA	60
85	E	CTX	63	T2N1	IIB	NST	Non-pCR	12	0	None	NA	60
86	E	CTX	46	T2N1	IIB	NST	Non-pCR	4	0	None	NA	60
26	F	PTMB	27	T2N0	IIA	NST	pCR	0	0	None	NA	60
47	F	PTMB	52	T2N1	IIB	NST	pCR	0	0	None	NA	60
16	F	CTX	64	T2N0	IIA	NST	pCR	0	0	None	NA	60
15	F	PTMB	43	T2N0	IIA	NST	pCR	0	0	None	NA	60
22	F	CTX	54	T2N0	IIA	NST	pCR	0	0	None	NA	60
27	F	PTMB	40	T2N0	IIA	NST	pCR	0	0	None	NA	60
39	F	CTX	48	T2N0	IIA	NST	pCR	0	0	None	NA	60
41	F	PTMB	30	T2N0	IIA	NST	pCR	0	0	None	NA	60
3	F	PTMB	41	T2N0	IIA	NST	pCR	0	0	None	NA	60
4	F	PTMB	31	T2N1	IIB	NST	pCR	0	0	None	NA	60
6	F	PTMB	65	T2N0	IIA	NST	pCR	0	0	None	NA	60
8	F	PTMB	36	T2N1	IIB	NST	pCR	0	0	None	NA	60
43	F	CTX	47	T2N0	IIA	NST	pCR	0	0	None	NA	60
77	F	PTMB	49	T2N1	IIB	NST	pCR	0	0	None	NA	60
78	F	PTMB	56	T3N0	IIB	NST	pCR	0	0	None	NA	60
79	F	CTX	61	T2N0	IIA	NST	pCR	0	0	None	NA	60
80	F	CTX	33	T2N1	IIB	NST	pCR	0	0	None	NA	60
81	F	PTMB	50	T2N0	IIA	NST	pCR	0	0	None	NA	60
82	F	PTMB	65	T2N1	IIA	NST	pCR	0	0	None	NA	60
83	F	PTMB	42	T2N0	IIA	NST	pCR	0	0	None	NA	60

Legend: *RT* residual tumor, *PTMB* panitumumab trial: panitumumab (Vectibix^®^), 9 mg/kg intravenous cycles combined with FEC × 4 (500/100/500 mg/m^2^), followed by docetaxel × 4 (100 mg/m^2^); CTX, cetuximab trial: 18 intravenous weekly infusions (Day1/8/15) of cetuximab (Erbitux^®^) (first infusion: 400 mg/m^2^; subsequently: 250 mg/m^2^) combined with docetaxel (100 mg/m^2^) on Day1 q3 weekly for six cycles; *NST* non-special type, *LNs* lymph nodes, *NAT* neoadjuvant treatment, *TTR* time to recurrence, pre, a sample taken before neoadjuvant treatment; post, a sample is taken after neoadjuvant treatment; *pCR* pathological complete response, *NA* not applicable.

Interpretable targeted panel sequencing (MSK-IMPACT) data were obtained for 45 patients, 26 from the PTMB trial and 19 from the CTX trial. For all patients who reached pCR (*n* = 13), only the pre-therapy sample was assessed, whereas pre-NAT and post-NAT matched samples were available in 17 of the 32 non-pCR patients. For 15 non-pCR patients, only the post-NAT sample was analyzed (Table 2).

**Table 2:** Samples used for molecular analyses and results of TIL amount assessment.

Pt ID	Group	Trial	Sample for IMPACT analysis	Samples for gene array (GA) and NanoString (NS) analysis	TILs (change of % from before to after treatment)
65	A	PTMB	Post	NS	10 → <1
49	A	PTMB	Post	GA + NS	1 → 5
9	A	PTMB	Pre + post	GA + NS	1 → 1
52	A	PTMB	Post	-	40 → 5
46	A	CTX	Pre + post	NS	< 1 → 5
71	A	PTMB	-	GA	10 → 10
38	B	PTMB	Pre	-	80 → NA (pCR)
10	B	PTMB	Pre + post	NS	< 1 → 5
33	B	CTX	Pre + post	NS	<1 → <1
44	B	CTX	Pre + post	NS	<1 → <1
72	B	PTMB	-	GA	100 → NA (pCR)
73	B	PTMB	-	GA	1 → NA (very small RT)
32	C	PTMB	Pre + post	GA + NS	10 → 5
60	C	CTX	Post	NS	<1 → <1
54	C	PTMB	Pre + post	GA	80 → 30
23	C	CTX	Pre + post	NS	70 → 30
50	C	PTMB	Pre + post	GA + NS	5 → 1
21	C	CTX	Post	GA + NS	2 → 40
11	C	PTMB	Pre + post	GA + NS	5 → 5
48	C	PTMB	Post	GA + NS	<1 → 5
68	D	PTMB	Post	GA	2 → 20
34	D	PTMB	Pre + post	GA + NS	40 → 20
45	D	CTX	Post	GA + NS	10 → 40
56	D	CTX	Post	-	<1 → 5
13	E	PTMB	Pre + post	GA	80 → 40
69	E	PTMB	Post	-	20 → 70
67	E	PTMB	Post	NS	30 → 20
64	E	CTX	Post	GA + NS	2 → 30
40	E	CTX	Pre + post	GA + NS	2 → 20
57	E	CTX	Post	GA + NS	1 → 1
70	E	CTX	Post	NS	<1 → 20
30	E	CTX	Pre + post	GA + NS	10 → 10
28	E	CTX	Pre + post	GA + NS	70 → 5
36	E	PTMB	Post	-	5 → 20
35	E	CTX	Pre + post	NS	100 → 100
19	E	CTX	Pre + post	-	40 → 40
74	E	PTMB	-	GA	50 → 50
75	E	CTX	-	GA + NS	10 → 20
76	E	PTMB	-	GA	90 → 90
84	E	PTMB	-	NS	2 → 90
85	E	CTX	-	NS	40 → 70
86	E	CTX	-	NS	30 → 90
26	F	PTMB	Pre	GA	20 → NA
47	F	PTMB	Pre	GA	2 → NA
16	F	CTX	Pre	GA	2 → NA
15	F	PTMB	Pre		100 → NA
22	F	CTX	Pre	GA	60 → NA
27	F	PTMB	Pre	GA	2 → NA
39	F	CTX	Pre	GA	90 → NA
41	F	PTMB	Pre	GA	40 → NA
3	F	PTMB	Pre	GA	40 → NA
4	F	PTMB	Pre	GA	30 → NA
6	F	PTMB	Pre	GA	80 → NA
8	F	PTMB	Pre	-	80 → NA
43	F	CTX	-	GA	100 → NA
77	F	PTMB	-	GA	40 → NA
78	F	PTMB	-	GA	5 → NA
79	F	CTX	-	GA	20 → NA
80	F	CTX	-	GA	40 → NA
81	F	PTMB	-	GA	90 → NA
82	F	PTMB	-	GA	100 → NA
83	F	PTMB	-	GA	30 → NA

*RT* residual tumor, *PTMB* panitumumab trial, *CTX* cetuximab trial, pre, sample taken before neoadjuvant treatment; post, sample taken after neoadjuvant treatment; *pCR* pathological complete response; *NA* not applicable. Samples for gene array and NanoString are pre- and post-therapy, respectively.

Gene expression analysis by gene arrays was performed in 41 pre-NAT samples, whereas NanoString technology was used to analyze 28 post-NAT samples (Table 2 and Supplementary Fig. 1).

### Tumor genomic features

**Fig. 1:**
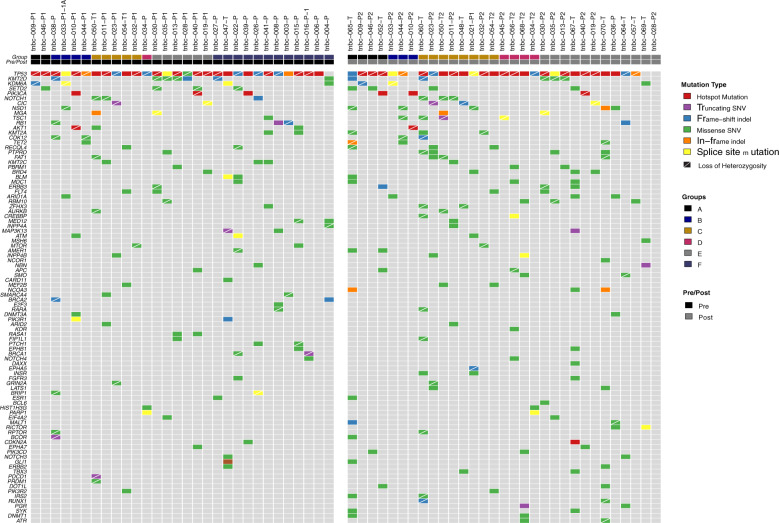
A heatmap depicting somatic mutations in 61 pre/post-treatment TNBC samples. Samples are shown as columns and are ordered by pre/post status and by the group. Genes that were altered in ≥2 samples are shown with colored squares corresponding to the colors in the legend. **A** rapidly recurring tumors (<1 year post-surgery); (**B**) tumors with standard recurrences (1–5 years after surgery); (**C**, **D**, and **E**) tumors without pathological complete response (pCR) to neoadjuvant treatment (NAT) and without recurrences; (**F**) tumors with pCR to NAT and without recurrences. Samples are presented by MSK-IMPACT assay identifiers (*X*-axis of the heatmap, identifiers of type tnbc-123-A or tnbc-123-AB).

**Fig. 2:**
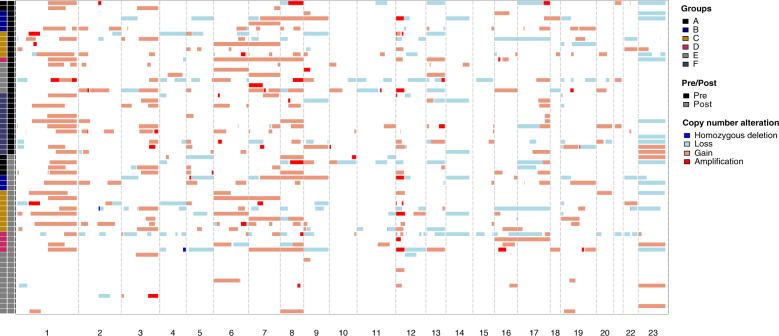
Copy number alterations in 61 pre/post-treatment TNBCs. A heatmap shows the amplifications, gains, losses, and homozygous deletions across all samples.

As expected, the vast majority of the tumors carried aberrations in *TP53* (44/45) (Fig. 1), which is consistent with the TNBC described genotype^24^. Interestingly, no mutations were found in one post-NAT sample from group A (patient 49, Table 1). In general, we observed copy number heterogeneity among the groups, with group F being an exception with very low copy number alterations (CNA) (Fig. 2), suggesting that genomic instability does not play a role in determining the aggressiveness of these tumors.

We observed a slight increase in mutations in the post-treatment samples, but this was not statistically significant (Fig. 1). Interestingly, among the 8 most mutated genes after *TP53*, 5 encode for epigenetic regulators: *KMT2D, KDM6A, SETD2, NSD1,* and *MGA*.

### Tumor gene expression profiles

**Fig. 3:**
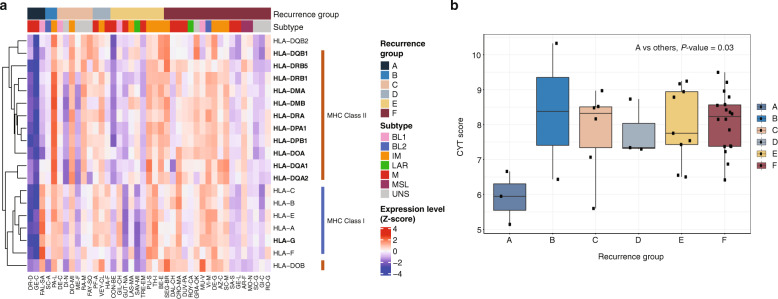
Molecular characteristics of the intratumor immune response. **a** Heatmap of gene expression in the HLA family. Differentially expressed genes identified from A vs. F are marked in bold. Z-score of expression level was calculated for each gene across all 41 patients. Samples are presented with their original institutional identifiers. A–F, recurrence groups; BL1, basal-like 1; BL2, basal-like 2, IM, immunomodulatory; LAR, luminal androgen receptor; M, mesenchymal; MSL, mesenchymal stem-like; UNS, unclassified. Samples are presented by MSK-IMPACT assay identifiers (X-axis of the heatmap, identifiers of type tnbc-123-A or tnbc-123-AB). **b** Immune cytolytic activity (CYT). *p*-value between group A tumors and others was calculated using a two-tailed Student’s *t*-test.

In the study of Masuda et al.^25^, TNBC subtypes are associated with patient outcomes. In our study, we classified patients into 6 subtypes suggested by Lehmann et al.^26^, based on gene array data (pre-treatment samples), to examine the enrichment of TNBC subtypes in our patient groups. However, we did not observe an enrichment of specific TNBC subtypes in any of our patient groups (Supplementary Fig. 2), suggesting that TNBC subtypes cannot be used to predict patient outcomes in this cohort. Further, there was no statistically significant difference in baseline gene expression between the tumors which recurred (groups A–B) and those which did not (groups C-F). However, differential gene expression analysis, comparing group A tumors with group F tumors (Supplementary Fig. 3), identified 12 HLA genes, which were significantly downregulated in group A. Moreover, all 6 MHC I class and 13 MHC II class HLA genes had lower expression in group A tumors (Fig. 3a), indicating that these tumors would elicit a low immune response. It should be noted that one of group A patients, patient 71 (Fig. 3a, Table 1, respectively), shows high expression in the MHC I class, presenting heterogeneity of HLA expression.

To confirm the low intratumor immune activity of group A tumors, we calculated the CYT score, a hallmark of cytolytic activity of the tumor immune infiltrate, as proposed by Rooney et al.^27^. As shown in Fig. 3b, group A tumors show significantly lower CYT scores (*P*-value <0.03, 95% CI: −3.72, −0.47) in comparison with the tumors with better prognosis, indicating that the intratumor immune activity in the group A tumors is indeed decreased, which might be one of the features permitting their rapid progression after therapy.

**Fig. 4:**
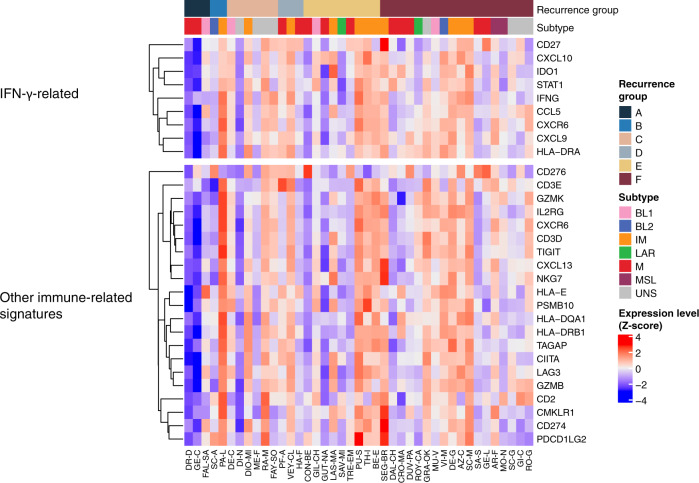
Expression of the IFN-γ-related and other immune genes. Z-score of expression level was calculated for each gene across all 41 patients. Samples are presented with their original institutional identifiers. A–F, recurrence groups; BL1 basal-like 1, BL2 basal-like 2, IM immunomodulatory, LAR luminal androgen receptor; M mesenchymal, MSL mesenchymal stem-like; UNS unclassified.

As IFN-γ is a key cytokine produced by the immune cells during the anti-tumor immune response, we examined the expression of the IFN-γ-related genes^28^ and found that, except for patient FAL-SA (patient 71, Table 1), another two patients in group A had extremely low baseline expression of many IFN-γ-related and other immune genes (Fig. 4). Together with low HLA gene expression and CYT scores, these findings reveal a “cold” immunological profile of the rapidly recurring tumors, which existed already before the NAT.

Through gene ontology (GO) enrichment analysis in “Molecular Function” terms, we observed 7 out of the top 10 enriched GO terms are related to downregulated cytokine and chemokine activities, including cytokine receptor activity (GO:0004896), cytokine binding (GO:0019955), cytokine activity (GO:0005125), cytokine receptor binding (GO:0005126), chemokine receptor binding (GO:0042379), and chemokine activity (GO:0008009) (Supplementary Fig. 4a). MHC protein complex binding (GO:0023023) is also enriched in downregulated genes, consistent with low HLA gene expression in group A tumors described above. Reactome pathway enrichment analysis identified enriched pathways in downregulated genes, for example, “chemokine receptors bind chemokines”, “interferon-gamma signaling”, “signaling by interleukins”, and so on (Supplementary Fig. 4b). Their pathway networks indicate that these pathways are immune-related (Supplementary Fig. 4c).

**Fig. 5:**
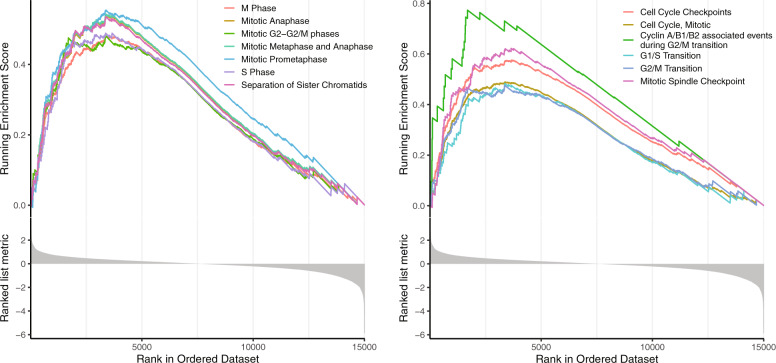
Representative gene set enrichment analysis plot showing upregulation of cell cycle-related genes. On *x*-axis, genes are ranked based on log_2_ fold change of gene expression level. From top to bottom, they are enrichment score of pathways on each gene, log_2_ fold change of expression level.

Another important difference in the baseline gene expression between group A and group F tumors was significantly higher expression of the cell cycle-related genes in group A. By gene set enrichment analysis we saw that the pathways enriched in those upregulated genes were “Polo-like kinase-mediated events”, “cyclin A/B1/B2 associated events during G2/M transition”, and “Chk1/Chk2(Cds1) mediated inactivation of Cyclin B: Cdk1 complex” (Supplementary Fig. 5). We further performed reactome gene set enrichment analysis using expression fold change values calculated from differential expression analysis between A and F and observed that cell cycle-related pathways are enriched in upregulated genes, including genes involved in different stages of the cell cycle (Fig. 5, left), for example, “S phase” and “separation of sister chromatids”, and genes involved in the regulation of cell cycle (Fig. 5, right), for example, “cell cycle checkpoints” and “mitotic spindle checkpoint”.

To examine genes related to metastasis, we compared gene expression data produced by the NanoString gene panel assessing genes involved in cancer progression, in post-NAT residues of tumors with and without recurrences. As shown in Supplementary Fig. 6, we observed that *SOX2*, a typical embryonic/stemness gene, and *CXCR4*, a well-known metastasis-related gene, are upregulated in group A tumors; *AREG*, encoding for amphiregulin, was upregulated in group B tumors. These data suggest that *SOX2*, *CXCR4*, and *AREG* may have a role in TNBC progression after NAT.

### Tumor-infiltrating lymphocytes (TILs)

In the RT of patients from group A, TILs were strikingly low before NAT ( ≤5% in all but one patient), which was concordant with gene expression showing low intratumor immune activity. Moreover, no patients in group A had increased TILs after NAT (Table 2). In group B, the baseline TIL density was also low (<1%) in 4 out of 6 evaluated cases. As in group A, no increase in TILs was observed after NAT, but 2 patients from this group reached pCR. Interestingly, one of the pCR cases had 100% TILs before NAT; however, she was diagnosed with a brain metastasis 23 months post-NAT and died a month after (patient 72, Table 2).

Most pCR cases had moderate or high amounts of TILs: 18 out of 22 cases had ≥20% of TILs before NAT, and 10 cases corresponded to the lymphocyte-predominant breast cancer (≥50% TILs) (Table 2). Two out of 23 pCR cases recurred; interestingly, both were very rich in TILs (80–100%) and both developed a brain metastasis, one of which was fatal.

These data indicate that very low TIL density (<5%), before NAT or in the post-NAT RT, could be an indicator of metastatic progression, however if associated with gene expression profile showing low anti-tumor immune activity, as seen in group A.

## Discussion

In this work, we present results obtained by molecular analyses of TNBC tissues sampled before and after NAT containing anti-EGFR antibodies and chemotherapy.

In search of molecular features associated with patient outcome, we first observed a marked heterogeneity of the analyzed samples in terms of mutation and CNA profile, without any specific alteration associated with metastatic recurrences. This is in line with other reports where they detect potentially actionable mutations in TNBC tissue but are unable to predict disease recurrence^6^. These findings may suggest that mutational profiling of TNBC is not the method of choice for the discovery of recurrence biomarkers and that other molecular analyses should be preferentially used for that purpose.

By analysis of gene expression, we distinguished a set of alterations characterizing TNBCs which progressed very rapidly after NAT completion. All but one of the six patients carrying those tumors died of metastatic disease in less than 6 months after surgery (data not shown). Rapid post-NAT progressors are relatively rare among TNBCs (3–5%, personal communication from Dr Mouret-Reynier, Centre Jean Perrin) and typically are undistinguishable from other non-responders in clinical trials. Our study showed that TNBCs with rapid metastatic progression after NAT containing anti-EGFR and chemotherapy had, already before therapy, two important biological characteristics: very low expression of several HLA class I or II genes and high expression of cell cycle-related genes.

MHC/HLA class I loss or downregulation is one of the main mechanisms of cancer immune escape, resulting in decreased T-cell cytolytic anti-tumor response^29^. In our series, the rapidly recurring tumors had both low expression of MHC I and -II genes and low cytolytic activity scores, indicating the absence of the anti-tumor immune reaction. In breast cancer in general, loss of HLA-I is associated with poor prognosis^30,31^. This was demonstrated also in TNBC, where higher expression of HLA class 2 molecules is linked to significantly longer disease-free survival^16,32^. One group has recently developed a 36-gene assay based on Nanostring technology, the MHCII Immune Activation Assay, which identifies TNBC patients with low risk of recurrence; patients with these tumors can be treated by immunotherapy instead of classical cytotoxic therapy^16^. Similarly, high expression of a gene signature containing *HLA-DRA*, *HLA-E*, *IDO1*, *CXCL9*, *CXCL10*, *STAT1,* and *GZMB* was found to be associated with TNBCs without relapse in 5 years after NAT^33^.

Besides low expression of the HLA genes, the rapidly recurring tumors in our series were characterized also by low expression of the IFN-γ signature and several other genes involved in the immune response within the tumor site. Most of those tumors had a very low amount of TILs before NAT and none of them increased TILs after NAT. This could explain their high resistance to the administered treatment, considered as capable of inducing immunogenic cell death and increasing tumor infiltration by cytolytic lymphocytes. Two recent studies proposed that the immune infiltration of post-NAT TNBC residues can influence the metastatic evolution of the disease. Patients with TNBC highly infiltrated by lymphocytes after NAT have longer metastasis-free survival than the ones having post-NAT residues with reduced amounts of TILs^34^. Moreover, low levels of post-NAT TILs in TNBC were shown to be associated with activation of the RAS/MAPK pathway, one of the proposed mechanisms of resistance to chemotherapy and metastatic progression of TNBC^35^. Thus, our data indicate that TNBC with very low TILs at the baseline and after NAT may represent a potentially aggressive category.

We also showed that the rapidly recurring tumors had a baseline expression of genes involved in the regulation of the cell cycle much higher than the tumors which experienced pCR to NAT and never recurred, but also higher than any other group in our cohort (data not shown). High pre-NAT proliferation, measured by the Ki67 index, has mostly been reported as a predictor of good response (pCR) to NAT;^36^ however, very high Ki67 indices (≥50%) were associated shown to be associated with progressive disease^37,38^. It has been postulated that activation of multiple receptor tyrosine kinases (RTKs) such as EGFR, IGF1R, or MET is responsible, at least in part, for the high proliferation of the progressive TNBCs^26^. In such cases, blockade of several RTKs might be more efficacious than single-type receptor blockade.

Our findings indicate that simultaneous assessment of the expression of genes involved in tumor proliferation and those involved in the immune response might be an effective way to stratify TNBC patients. In a series of 1954 breast cancers of all molecular subtypes, high expression of a proliferation-related signature and of all three immune metagenes was associated with longer distant metastasis-free survival (DMFS). However, in those highly proliferative breast cancers, low expression of any of the immune metagenes was associated with short DMFS despite high expression of the other two^39^. These data support the high importance of strong immune control of the highly proliferating breast cancers for a good outcome. On the other hand, the association of high proliferation and immunological silence would favor rapid tumor progression, as observed in our patient cohort.

Our analysis of the post-NAT residues, although very limited, highlighted two pro-metastatic genes, *SOX2* and *CXCR4*, as potential recurrence predictors. *SOX2* has been shown to promote proliferation and metastasis in TNBC^40^. Similarly, several previous studies have shown an association between high *CXCR4* expression and breast cancer metastasis^41^. Interestingly, a recent study showed a strong correlation between high *CXCR4* expression and high TIL count^42^, whereas in our study high *CXCR4* expressors were devoid of TILs. More work is therefore necessary to fully explore the relationship between *SOX2*, *CXCR4,* and the immune microenvironment of TNBC, however these three parameters seem to have the capacity to predict patient outcomes in TNBC.

Besides potential biomarkers of rapid recurrence after NAT, we did not reveal biomarkers that could stratify the entire cohort into prognostic groups. This likely can be explained by a small number of recurrences as well as of patients/samples analyzed and the heterogeneity of the cohort. These characteristics of the cohort we analyzed here are surely the major limitations of the study. However, since the trials we conducted were the first trials of neoadjuvant EGFR blockade in TNBC, we wanted to analyze the available tissue material, even limited, to generate at least some hypotheses. Another limitation of this study was our inability to calculate residual cancer burden^43^ because of the lack of the necessary material in some surgical specimens (due to the multicentricity of the clinical trials). It would have been interesting to see whether any of the evaluated molecular features had a specific relationship with any of the RCB categories.

In conclusion, we found that TNBC with high baseline expression of cell cycle genes, associated with a “cold” immune microenvironment, may have a very poor prognosis following neoadjuvant treatment containing chemotherapy and anti-EGFR antibodies. Therefore, high pre-treatment proliferation and low intratumor immune response are worth evaluation in larger studies, on cohorts treated by various neoadjuvant treatments, to verify their general and treatment-specific prognostic value.

## Methods

### Patients and tumor samples

The available material (formalin-fixed, paraffin-embedded (FFPE) tumor tissues or extracted DNA) from the patients enrolled in clinical trials NCT00933517 (panitumumab trial, PTMB) and NCT00600249 (cetuximab trial, CTX) was used. Details on the inclusion criteria, treatment, response, and non-genomic predictive biomarkers are previously published^22,23^. The clinical follow-up of 5 years after surgery ended in August 2017. Recurrence was defined as the appearance of any invasive cancer deposit. All local and most distant recurrences were histologically confirmed, except brain metastases which were diagnosed by imaging.

The greatest dimension of post-NAT breast residual tumor and the number of involved axillary lymph nodes were retrieved from the pathology reports received from the participating centers in the trials. The tumors were all but one of Scarff-Bloom-Richardson (SBR) grade 3. The only tumor of SBR grade 2 was an apocrine carcinoma in group E. No SBR grade change was observed after the NAT, in comparison to the pre-NAT grade.

Quantity of tumor-infiltrating lymphocytes (TILs) was estimated on H&E-stained FFPE pre-NAT and post-NAT tumor tissue sections, according to the recommendations of the International Immuno-Oncology Biomarker Working Group on Breast Cancer^44,45^.

### Targeted exome sequencing

DNA samples extracted from formalin-fixed, paraffin-embedded TNBCs (*n* = 62) were subjected to MSK-IMPACT targeted sequencing at the MSKCC Integrated Genomics Operation as previously described^46^. Briefly, raw sequence reads were aligned to the reference human genome GRCh37 using the BURROWS-WHEELER ALIGNER (BWA 0.7.15)^47^. Local realignment, duplicate read removal, and base quality score recalibration were performed using the GENOME ANALYSIS TOOLKIT (GATK 3.7)^48^. Somatic single nucleotide variants (SNVs) were called using MUTECT (1.1.7)^49^, and small insertions and deletions (indels) were identified using STRELKA (1.0.15)^50^, VARSCAN2 (2.3.7)^51^, LANCET (1.0.0)^52^, and SCALPEL (0.5.3)^53^ and further curated by manual inspection. SNVs and indels outside of target regions were filtered out, as were SNVs and indels for which the variant allele fraction (VAF) in the tumor sample was <5 times that of the paired normal VAF as previously described^46,54^. Finally, SNVs and indels found at >5% global minor allele frequency in dbSNP (build 137) and >5% global allele frequency in EXAC (0.3.1) were discarded. Somatic copy number alterations and loss of heterozygosity were obtained using FACETS^55^. The cancer cell fractions (CCF) of all mutations were computed using ABSOLUTE (1.0.6)^56^. A mutation was classified as clonal if its probability of being clonal was >50%^57^ or if the lower bound of the 95% confidence interval of its CCF was >90%^46,54^. Mutations that did not meet the above criteria were considered subclonal. A combination of in silico functional predictors was used to define the potential functional impact of each missense SNV as previously described^46,58^. Mutation hotspots were assigned according to Chang et al.^59^.

### Gene expression by gene arrays

Nucleic acids were extracted from frozen tumor tissue (pre-NAT samples) using AllPrep DNA/RNA mini kit (Qiagen France SAS, Courtaboeuf, France) according to the manufacturer’s protocol. RNA quality was verified using the 2100 BioAnalyzer (Agilent Technologies). The extracted material was sent to Helixio (Saint-Beauzire, France), where it was hybridized with gene arrays (Human SurePrint, Agilent Technologies France, Les Ullis, France). The raw data were transferred to Memorial Sloan Kettering Cancer Center for bioinformatics and statistical analysis.

Limma package^60^ was applied to process Agilent microarray data and identify differentially expressed genes between progressive tumors and tumors with pathological complete response. Background correction was performed on expression intensities before normalization by the parameter of “expnorm”. The median value was taken when a gene has multiple probes. Then, “cyclicloess” method, which applies loess normalization to all possible pairs of arrays, was used to normalize corrected intensities. Fold change of expression intensities and P-values were estimated, which were used to identify differentially expressed genes.

We performed GO^61^ and Reactome pathway^62^ enrichment analysis with clusterProfiler^63^ and ReactomePA^64^. Over-representation analysis and gene set enrichment analysis were performed. Benjamini-Hochberg method was used to calculate adjusted p-values.

TNBCtype was used to classify samples into transcriptomic subtypes^26,65^.

Immune cytolytic activity of the local immune infiltrate was quantified by measuring expression of the granzyme A (*GZMA*) and perforin (*PRF1*) genes, as proposed by Rooney et al.^27^. We calculated the CYT score by averaging the expression level of *GZMA* and *PRF1* for each microarray sample^66^.

### Gene expression by NanoString

RNA was isolated from FFPE samples of post-NAT residual tumors using the High Pure FFPET RNA Isolation Kit (Roche Diagnostics, Meylan, France), according to the manufacturer’s instructions. RNA purity and concentration were determined using a spectrophotometer (BioPhotometer, Eppendorf). Only the samples with a purity of ≥1.6 were used. One hundred nanograms of RNA was loaded for hybridization with nCounter^®^ PanCancer Progression Panel, according to the manufacturer’s instructions (NanoString, Seattle, WA, USA). The hybridization signals were analyzed by the nCounter^®^ FLEX Analysis System (NanoString).

Raw count data, which include 740 endogenous genes, 6 positive controls, 8 negative controls, and 30 housekeeping genes, were preprocessed using the R package NanoStringNorm^67^. Raw counts were merged by patients by taking an average of samples. Specifically, geometric mean-based normalization was used to normalize for technical assay variation, followed by background adjustment based on negative controls, where “mean.2sd” is used. RNA content normalization was performed by all 30 housekeeping genes with the parameter “housekeeping.geo.mean”. Finally, log2-transformed data were used for downstream analysis. One sample was excluded for the analysis due to the high positive normalization factor examined by NanoStringNorm^67^. Differential expression analysis was performed using the R package Limma^60^.

### Statistical analysis

The statistical significance of CYT differences between group A and other tumors was evaluated by a two-tailed Student *t* test in the R package.

### Ethical approval

This study was declared to and approved by the French National Commission for Informatics and Freedom (Commission Nationale de l’Informatique et des Libertés), under number 1209138, as well as by the Ethical Committee of Clermont Ferrand (numbers AU 806 and 711). Each patient signed an informed consent presenting details of the study.

### Reporting summary

Further information on research design is available in the Nature Research Reporting Summary linked to this article.

## Supplementary information

Supplementary Information

Reporting Summary

## Data Availability

The assembled prospective somatic mutational data from ctDNA and tumors for the entire cohort have been deposited for visualization and download in the cBioPortal for Cancer Genomics (http://cbioportal.org/). MSK-IMPACT DNA sequencing data is available under SRA accession code PRJNA750756, gene array RNA sequencing data is available under GEO accession code GSE180775 and nanostring sequencing data is available under GEO accession code GSE180717. All other data supporting the findings of this study are available from the corresponding author on reasonable request.
