# Supplementary material for: Recurrence biomarkers of triple negative breast cancer treated with neoadjuvant chemotherapy and anti-EGFR antibodies
Source: NPJ Breast Cancer. 2021 Sep 17;7:124. doi: 10.1038/s41523-021-00334-5 (PMC8448841; doi:10.1038/s41523-021-00334-5)
Supplement: Supplementary file 1 — Supplementary Information [file 41523_2021_334_MOESM1_ESM.pdf]

Supplementary Figure 1

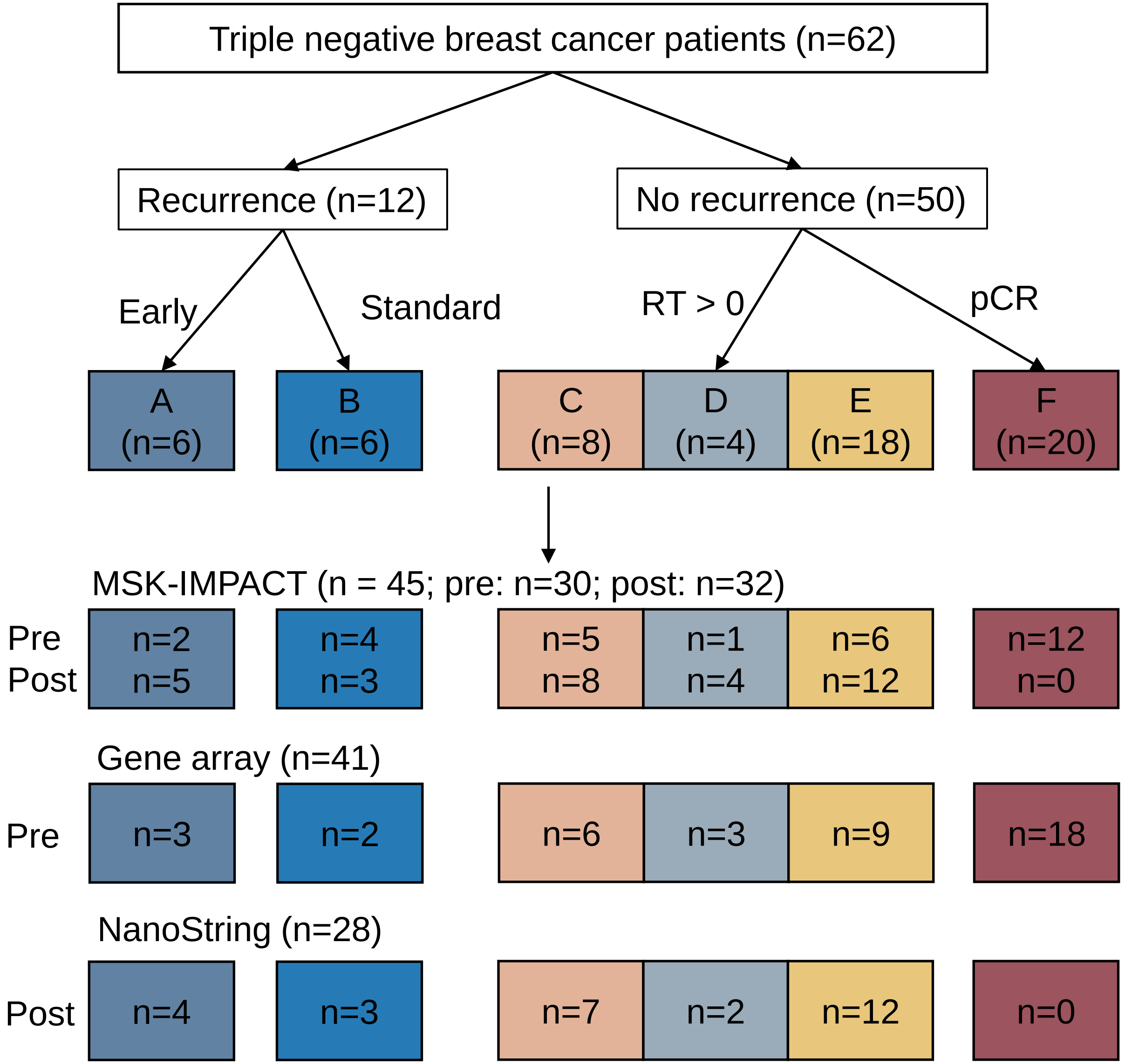

Legend: Up-regulated genes involved in cell cycle pathways identified by differential expression analysis in the comparisons of group A vs. group F tumors. Reactome pathways enriched in up-regulated genes in comparison of group A with group F. Benjamini-Hockberg method was used to calculate adjusted p-value.

Supplementary Figure 2

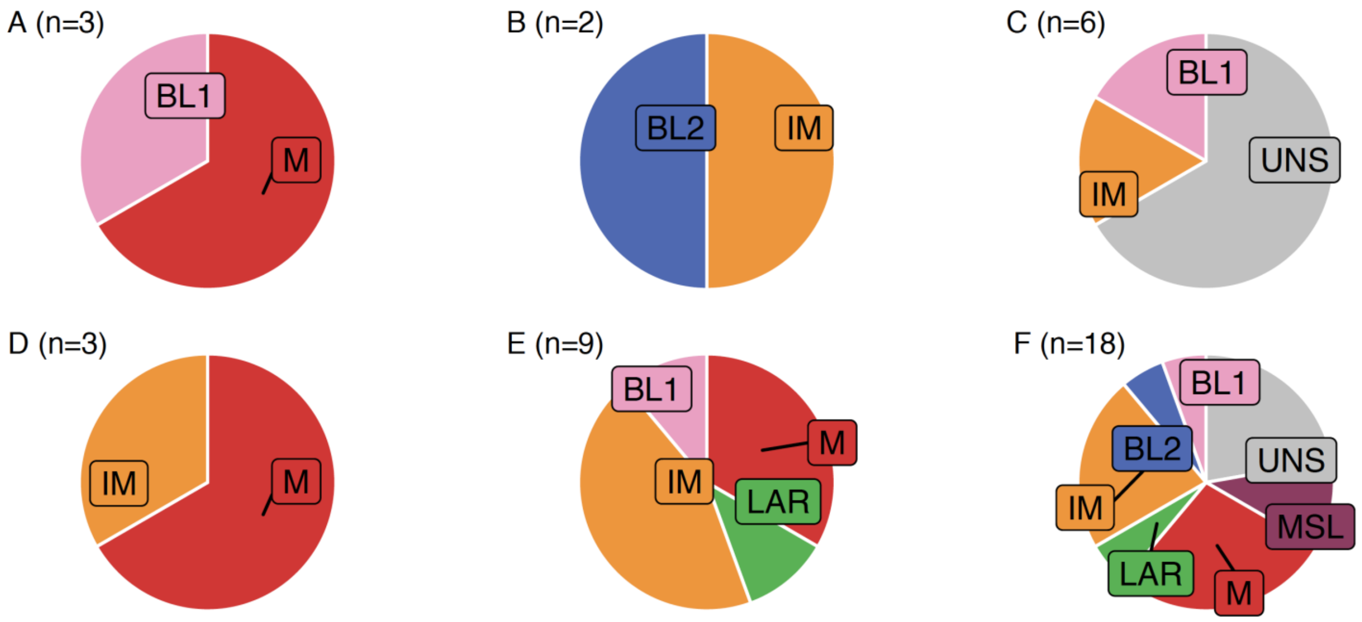

Legend: TNBC subtypes based on gene array data and TNBCtype use.
BL1, basal-like 1; BL2, basal-like 2; IM, immunomodulatory; M, mesenchymal; MSL, mesenchymal stem–like; LAR, luminal androgen receptor; UNS, unclassified.

Supplementary Figure 3

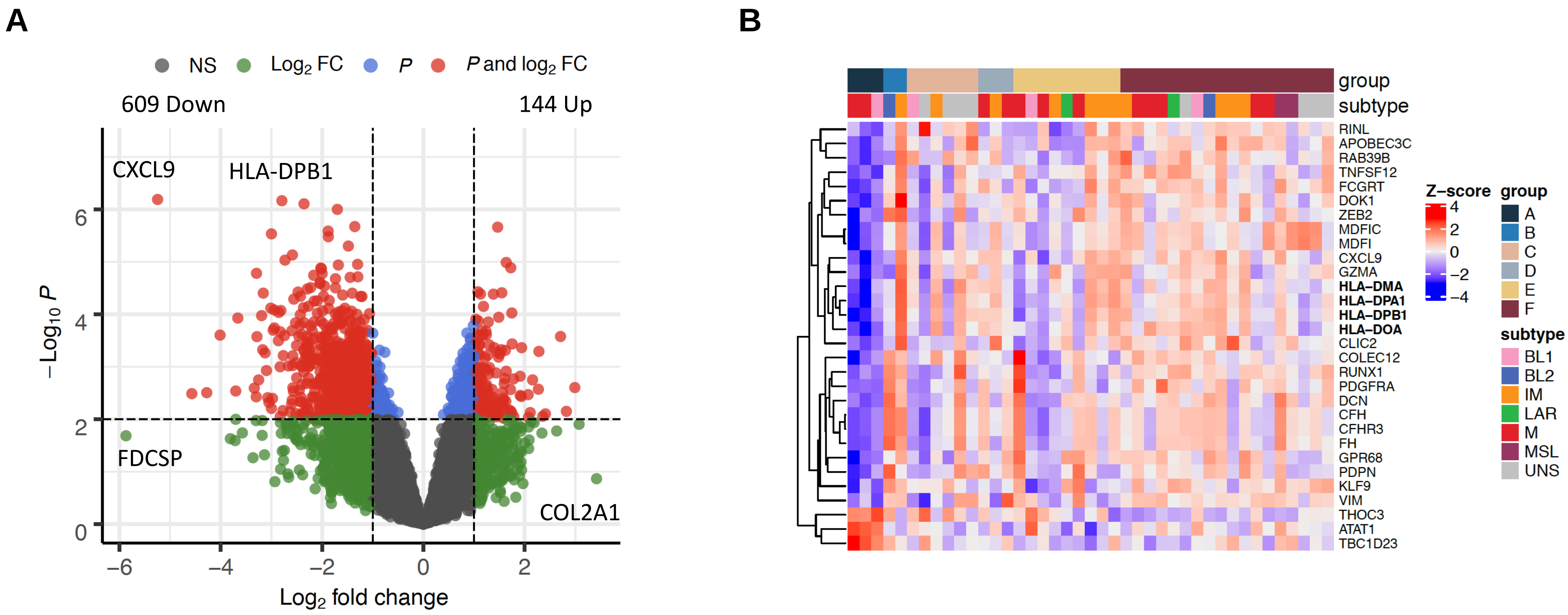

Legend: Differentially expressed genes in the comparisons of group A vs. group F tumors.
A) Volcano plot indicating the presence of differentially expressed genes between group A and group F tumors;
B) Heatmap of gene expression level for top 30 differentially expressed genes in comparison of group A with group F tumors.
Z-score was calculated for each gene by expression level.

Supplementary Figure 4

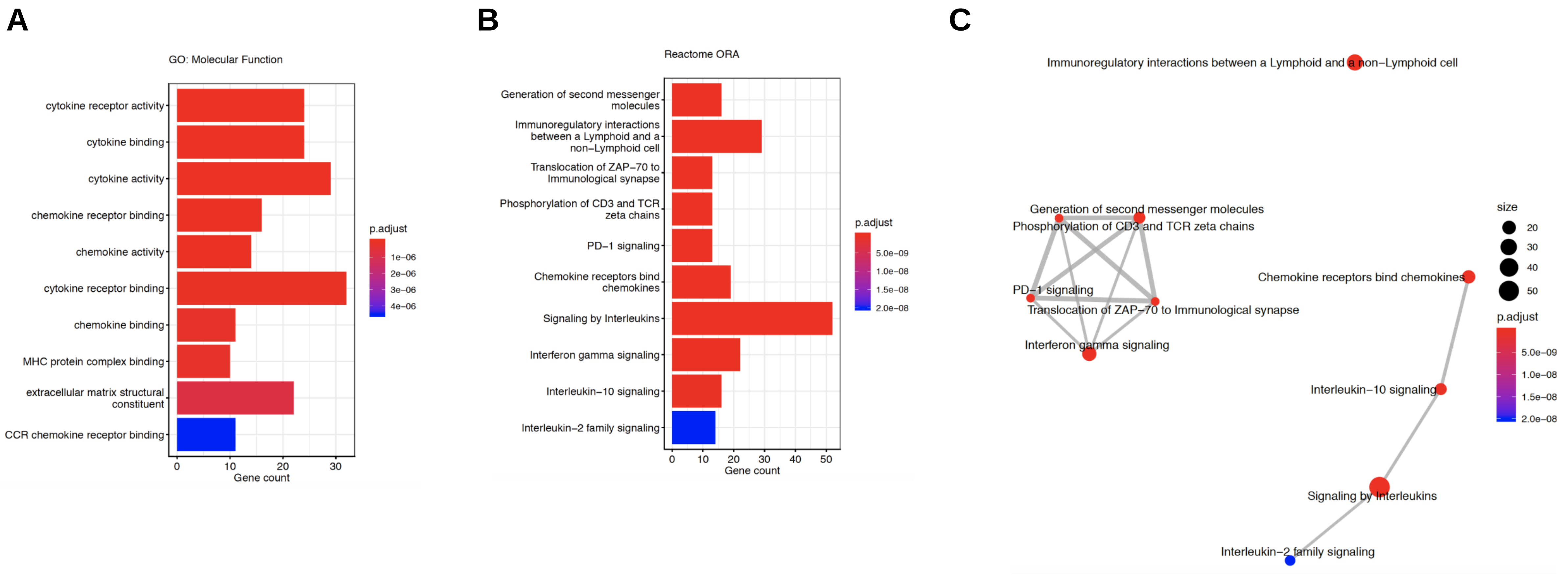

Legend: Activities of cytokine and chemokine receptors down-regulated in group A tumors.

A) Top 10 gene ontologies in “Molecular Function” enriched in down-regulated genes of group A;

B) B) Top 10 Reactome pathways enriched in down-regulated genes of group A;

C) Reactome pathway network. Nodes represent Reactome pathways, edges are the number of genes shared by pathways.

Benjamini-Hockberg method was used to calculate adjusted p-value.

Supplementary Figure 5

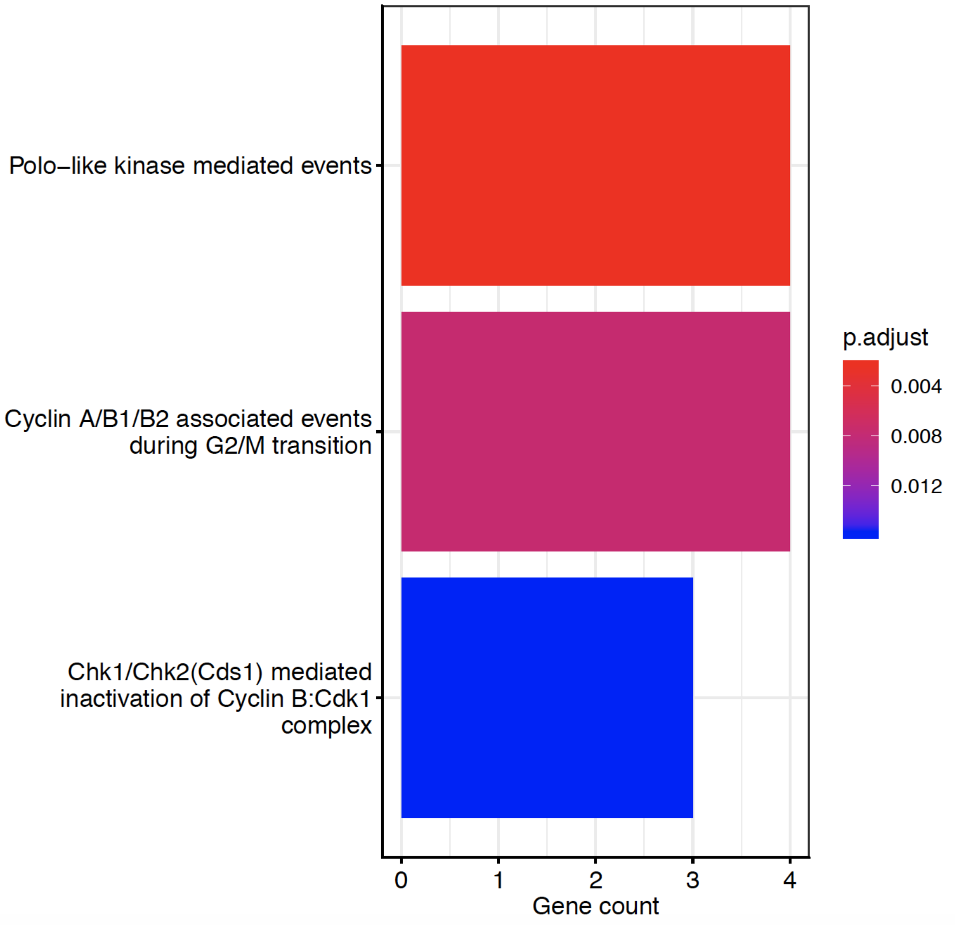

Legend: Up-regulated genes involved in cell cycle pathways identified by differential expression analysis in the comparisons of group A vs. group F tumors. Reactome pathways enriched in up-regulated genes in comparison of group A with group F tumors. Benjamini-Hockberg method was used to calculate adjusted p-value.

Supplementary Figure 6

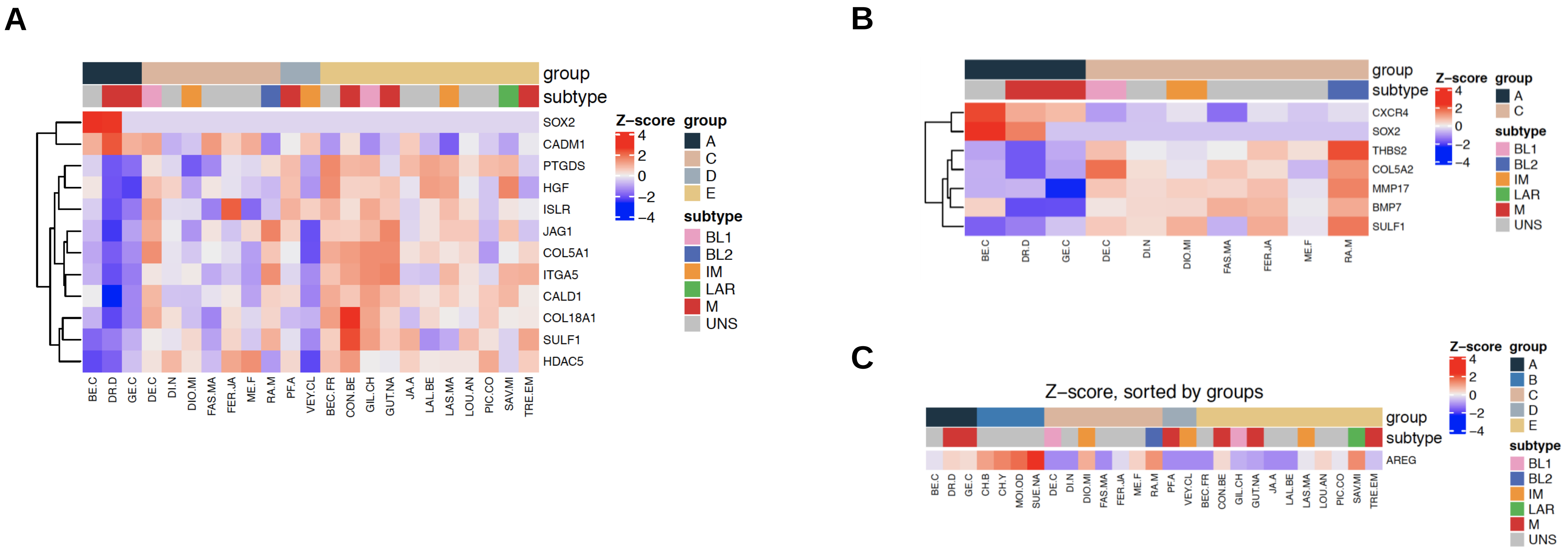

Legend: Putative biomarkers of metastatic recurrence identified by analysis of gene expression in post-treatment residual tumors using NanoString PanCancer Progression Panel.

A) Differentially expressed genes in the comparisons of group A (rapid fatal recurrence) vs. merged groups C, D and E (no recurrence);

B) B) Differentially expressed genes in the comparisons of group A (rapid fatal recurrence) vs. group C (no recurrence despite big post-treatment residues, > ypT1c);

C) C) Differentially expressed genes in the comparisons of merged groups A (rapid fatal recurrence) and B (standard, non-fatal recurrence) vs. merged groups C, D and E (no recurrence).

Samples are presented by patient clinical identifiers (X-axis of the heatmaps, identifiers of type AB-C or ABC-DEF).
