# Supplementary material for: Recurrence biomarkers of triple negative breast cancer treated with neoadjuvant chemotherapy and anti-EGFR antibodies
Source: NPJ Breast Cancer. 2021 Sep 17;7:124. doi: 10.1038/s41523-021-00334-5 (PMC8448841; doi:10.1038/s41523-021-00334-5)
Supplement: Supplementary file 2 — Reporting Summary [file 41523_2021_334_MOESM2_ESM.pdf]

nature research

Corresponding author(s): Maurizio Scaltriti
Jorge Reis-Filho

Last updated by author(s): Aug 17, 2021

Reporting Summary

Nature Research wishes to improve the reproducibility of the work that we publish. This form provides structure for consistency and transparency in reporting. For further information on Nature Research policies, see our Editorial Policies and the Editorial Policy Checklist.

Statistics

For all statistical analyses, confirm that the following items are present in the figure legend, table legend, main text, or Methods section.

n/a	Confirmed	
☐	☒	The exact sample size (n) for each experimental group/condition, given as a discrete number and unit of measurement
☐	☒	A statement on whether measurements were taken from distinct samples or whether the same sample was measured repeatedly
☐	☒	The statistical test(s) used AND whether they are one- or two-sided<br>*Only common tests should be described solely by name; describe more complex techniques in the Methods section.*
☐	☒	A description of all covariates tested
☐	☒	A description of any assumptions or corrections, such as tests of normality and adjustment for multiple comparisons
☐	☒	A full description of the statistical parameters including central tendency (e.g. means) or other basic estimates (e.g. regression coefficient) AND variation (e.g. standard deviation) or associated estimates of uncertainty (e.g. confidence intervals)
☐	☒	For null hypothesis testing, the test statistic (e.g. F, t, r) with confidence intervals, effect sizes, degrees of freedom and P value noted<br>*Give P values as exact values whenever suitable.*
☒	☐	For Bayesian analysis, information on the choice of priors and Markov chain Monte Carlo settings
☒	☐	For hierarchical and complex designs, identification of the appropriate level for tests and full reporting of outcomes
☒	☐	Estimates of effect sizes (e.g. Cohen's d, Pearson's r), indicating how they were calculated

Our web collection on statistics for biologists contains articles on many of the points above.

Software and code

Policy information about availability of computer code

Data collection	No software was used.
Data analysis	Raw sequence reads were aligned to the reference human genome GRCh37 using the BURROWS-WHEELER ALIGNER (BWA 0.7.15).<br>Local realignment, duplicate read removal, and base quality score recalibration were performed using the GENOME ANALYSIS TOOLKIT (GATK 3.7)4.<br>Somatic single nucleotide variants (SNVs) were called using MUTECT (1.1.7), and small insertions and deletions (indels) were identified using STRELKA (1.0.15), VARSCAN2 (2.3.7), LANCET (1.0.0), and SCALPEL (0.5.3) and further curated by manual inspection. SNVs and indels outside of target regions were filtered out, as were SNVs and indels for which the variant allele fraction (VAF) in the tumor sample was < 5 times that of the paired normal VAF as previously described. Finally, SNVs and indels found at > 5% global minor allele frequency in dbSNP (build 137) and > 5% global allele frequency in EXAC (0.3.1) were discarded. Somatic copy number alterations and loss of heterozygosity were obtained using FACETS. The cancer cell fractions (CCF) of all mutations were computed using ABSOLUTE (1.0.6).

For manuscripts utilizing custom algorithms or software that are central to the research but not yet described in published literature, software must be made available to editors and reviewers. We strongly encourage code deposition in a community repository (e.g. GitHub). See the Nature Research guidelines for submitting code & software for further information.

April 2020

Data

Policy information about availability of data

All manuscripts must include a data availability statement. This statement should provide the following information, where applicable:

- Accession codes, unique identifiers, or web links for publicly available datasets
- A list of figures that have associated raw data
- A description of any restrictions on data availability

The assembled prospective somatic mutational data from ctDNA and tumors for the entire cohort have been deposited for visualization and download in the cBioPortal for Cancer Genomics (http://cbioportal.org/).
All other data supporting the findings of this study are available from the corresponding author on reasonable request.

Field-specific reporting

Please select the one below that is the best fit for your research. If you are not sure, read the appropriate sections before making your selection.

☒ Life sciences ☐ Behavioural & social sciences ☐ Ecological, evolutionary & environmental sciences

For a reference copy of the document with all sections, see nature.com/documents/nr-reporting-summary-flat.pdf

Life sciences study design

All studies must disclose on these points even when the disclosure is negative.

Sample size	The study was performed on all available tissue samples from patients enrolled in clinical trials NCT00933517 and NCT00600249. The study was performed as an exploratory, hypothesis-generating study, so no sample size calculation was done.
Data exclusions	N/A
Replication	The reproducibility of the data presented is included in either Results or Materials and Methods of the manuscript.
Randomization	The randomization is described in either Results or Materials and Methods of the manuscript.
Blinding	Investigators were blinded to groups allocation.

Reporting for specific materials, systems and methods

We require information from authors about some types of materials, experimental systems and methods used in many studies. Here, indicate whether each material, system or method listed is relevant to your study. If you are not sure if a list item applies to your research, read the appropriate section before selecting a response.

Materials & experimental systems

n/a	Involved in the study	
☒	☐	Antibodies
☒	☐	Eukaryotic cell lines
☒	☐	Palaeontology and archaeology
☒	☐	Animals and other organisms
☒	☐	Human research participants
☐	☒	Clinical data
☒	☐	Dual use research of concern

Methods

n/a	Involved in the study	
☒	☐	ChIP-seq
☒	☐	Flow cytometry
☒	☐	MRI-based neuroimaging

Clinical data

Policy information about clinical studies

All manuscripts should comply with the ICMJE guidelines for publication of clinical research and a completed CONSORT checklist must be included with all submissions.

Clinical trial registration	Clinical trials NCT00933517 and NCT00600249.
Study protocol	The full trial protocol can be found in the following publications: Nabholtz JM et al., Ann Oncol 2014, PMID 24827135 and Nabholtz et al., Int J Cancer 2016, PMID 26649807
Data collection	The clinical data used in this study (the presence of metastatic recurrences, the date of the first metastatic recurrence, the death date and the patient status (alive/deceased) were extracted from a database containing all the data from clinical trials NCT00933517

and NCT00600249. The database is localized at the Centre Jean Perrin, Clermont-Ferrand, France (the institution which was the sponsor of the trials).

Outcomes	The outcome measures were: the presence of metastatic recurrences, the time to the first metastatic recurrence and the patient status (alive/deceased).<br>The presence of a metastatic recurrence was confirmed by biopsy or by imaging (positron-emission tomography or magnetic resonance imaging).<br>The time to the first metastatic recurrence was calculated in months, from the breast surgery date to the date of diagnosis of the first metastatic recurrence.<br>The patient status (alive/deceased) was assessed five years after the breast surgery date, by interrogation the patients about their health status (written addressing to the patient and to her primary physician/general practitioner). The data about patients' death were collected from death certificates issued by the authorities which confirmed the death.
